# Notes on the Methods of Performing Paracentesis of the Various Cavitives

**Published:** 1894-09-29

**Authors:** John Poland

**Affiliations:** Surgeon to the Miller Hospital, Greenwich


### NOTES ON THE METHODS OF PERFORMING PARACENTESIS OF THE VARIOUS CAVITIES.

By John Poland, F.R.C.S., Surgeon to the Miller Hospital, Greenwich.

Although no new apparatus or instruments for evacuating the fluid contents of cavities are introduced in this paper, those employed at the present time being in reality the same as those in use some twenty or thirty years ago, yet the altered conditions under which each is used (owing partly to our increased knowledge of the pathology of the diseases in question), the modifications these instruments have undergone, and their employment with antiseptic precautions, have produced such a marked improvement in the mortality of these affections that I believe this subject is one of the greatest practical importance. We may with advantage dismiss tapping of cysts or abscesses in the liver, ovarian and kidneys cysts, pus or fluid in joints, and other like conditions, confining ourselves to the cavities of the body.

The more simple forms of instruments are the best: (1) Aspiration by Dieulafoy's or Potain's instrument, (2) the more rapid evacuation by trocars and cannulas of varying size, (3) subaqueous syphon drainage, (4) drainage by Southey's "tubes," and (5) permanent syphon drainage are all in vogue at the present time. The employment of each method will be treated under the different diseases. Taking the pleural cavity first, how should we treat: 1. Pleurisy with effusion. 2. Empyema. 3. Pyo-pneumothorax.

Operations upon the thorax for the relief of effusion into the pleural cavities have been practised from the time of Hippocrates downwards. Galen performed aspiration. But until very recent times, before the introduction of antiseptic and aseptic methods. Thoracentesis, or tapping the chest, was regarded as an operation of great danger. It was hardly ever performed, except for cases of empyema about to point externally. Thoracentesis may be employed with safety from infancy up to advanced age. As a rule, the risk increases slightly after thirty. It may be very successfully used in all simple serous effusions, in pleural effusion during pregnancy, and during the puerperal state and cardiac disease. The effusion of rheumatic fever is usually rapidly absorbed without aspiration; but it is sometimes called for in exceptional cases with urgent symptoms. Aspiration is only a temporary measure in the effusion of chronic Bright's disease, and its employment in advanced phthisis is very questionable. Tapping should be performed in large effusions of a quiet chronic character, *i.e.*, the cavity should be at least two-thirds full, and of two or three weeks' duration. When effusion has existed for any length of time the pleura becomes accustomed to the condition, and absorption does not take place; the fluid should, therefore, not be permitted to remain in the pleural sac, at any rate longer than the three weeks; while, no doubt, the natural tendency for uncomplicated pleuritic effusion in a large number of cases is to clear up, on the other hand the operation should not be delayed till the lung has become compressed or bound down by adhesion. Although we generally wait till the inflammatory stage or acute febrile condition has subsided, it must be performed where there is danger to the vitality of the lung, in bilateral pleurisy, danger of cardiac syncope, extreme dyspnœa, orthopnœa, or cyanosis. A steady increase of the effusion, especially if it is on the left side, signs of pressure, or congestion of the opposite lung, are also indications.

As to the operation itself, Potain's aspirator is a very perfect instrument of the kind, and is preferable to Dieulafoy's; the latter is liable by its pointed end to injure the lung, and is more useful for diagnosis. In Potain's or Thompson's aspirator facilities are afforded for clearing the tube of any obstruction. A blunt plug or pilot trocar may be passed down and the cannula cleared without any air entering; a trocar in the outer tube is always preferable. These are advantages which we do not get in Dieulafoy's instrument. The diameter of the tube should be $\frac{1}{12}$ to $\frac{1}{8}$ inch; a tube of this size is preferable to the very small ones often recommended, as there is almost the same pain in introducing either, and the former is not so liable to be plugged or broken.

The instruments and skin should be thoroughly carbolised with 1 in 20 carbolic lotion, not oil. The needle or trocar and cannula should be thoroughly disinfected and cleansed, and some lotion 1 in 20 should be allowed to pass through the tube before using, to see that all is in working order as well as to cleanse the instrument. The hands of the surgeon should be scrupulously clean. If these precautions be not taken a simple serous effusion may be changed into a purulent one, or even something worse. The skin of the patient may be rendered insensitive, either by the anestile spray (Dr. Bengué), by the ether spray, or by ice and salt. A strong solution of carbolic acid, applied to the skin for about three minutes, is another excellent local anæsthetic; for many years I have used it in this way in various small operations on the skin, removal of small tumours, &c. It has a penetrating power affecting the whole thickness of the skin besides being an antiseptic agent.

The patient is then placed in a slightly recumbent position and lying on the sound side for fear of faintness, with the arm well elevated; children may be in a sitting position in the arms of a nurse. Firm pressure is made with the index finger of the left hand in the intercostal space close to the trocar, in order to avoid striking against a rib, which may happen if the ribs are close together. The vacuum should be turned on directly the needle is through the skin. The trocar is then passed close to the upper border of the lower rib to avoid the intercostal vessels which run along the lower border of the ribs, and plunged rapidly so as to puncture the thick pleura or lymph; this might otherwise be separated from the ribs, and pushed before the trocar. The site of puncture should be the fourth or fifth interspace on the right side and the sixth or seventh on the left in the anterior axillary line, or just in front of the posterior fold of the axilla, or the seventh, eighth, or ninth space behind, one or two inches outside the angle of the scapula. In children, when the effusion is on the right side, the puncture should not be made lower than the fourth or fifth interspace as the liver rises higher in them than in adults.

The fluid should not be drawn off quickly, it should be done slowly for fear of a vessel being ruptured and hæmothorax produced. It is best to continue the exhaustion of the aspiration bottle during the flow, so that the successive vacuums may be absent and too great force of pressure upon the lung avoided. Two years ago I aspirated the left pleural cavity of a young woman between the fifth and sixth month of pregnancy. The heart was much displaced with intense dysnœa. Aspiration was very slowly performed, the operation lasting an hour and a half, and fifty-one ounces of fluid removed. It was followed by great relief and complete recovery. The gradual relief of the urgent symptoms in this case was very noticeable during the slow removal of the fluid, and very gratifying to the patient.

It is unnecessary in these cases to remove all the fluid—two pints is often enough. Absorption frequently takes place rapidly after the removal of the intrathoracic pressure, even if it be a large effusion which had previously been stationary. As a matter of fact it is quite impossible to remove *all* fluid from a chest by this means. Immediately desist from further aspiration if the pulse becomes bad, the patient faint, or cough or pain produced, or blood appears in the fluid from giving way of some vessel in the lung. On withdrawing the cannula it is well for the patient to hold his breath, to avoid inspiration and air entering at the seat of puncture. The puncture should be closed with collodion and the side carefully bandaged.

Thus the dangers of thoracentesis are syncope from sudden removal of the whole of the fluid, which had displaced the heart and large vessels, and acute congestion or œdema of the lungs.

Slight reaccumulations are often reabsorbed; the indications for their removal are the same as for a first aspiration. It is not an uncommon thing to see absorption take place after one or two aspirations.

Aspiration or the use of a vacuum is not a necessity for the removal of fluid; the elasticity of the thorax is sufficient to expel the fluid through a trocar and syphon tube. The lower end of the tube being immersed in antiseptic solution, the aspirator may be used as a syphon if a stop-cock and tube be attached between the cannula and bottle. The chest may by this means be emptied very effectively. Large trocars and cannulas are apt to get loose and admit air; they must be used, however, if there is much lymph.

The objects of aspiration may be briefly stated—(1) Relief or prevention of urgent symptoms and possible death from effects of pressure of rapid or excessive effusions. In dyspnœa or orthopnæa delay is always dangerous. (2) Duration of disease is shortened. By a simple aspiration fluid can be removed in a short time which nature could only do in four weeks or more. (3) Prevention of the effusion becoming chronic by repeated tappings, for we know how grave an affection chronic pleuritic effusion becomes if left. (4) To obviate the possibility of the collapsed lung becoming inexpansible. In short, the danger of delay is greater than the danger incurred in doing it too early. Finally, one word of precaution—An exploratory puncture with a needle should always precede thoracentesis, on account of the insufficient data for distinguishing serous from purulent collections of fluid, and even solid conditions of the lungs and pleuræ in children.

(1) Absolute dulness on percussion, (2) absence of respiratory sounds, (3) absence of vocal fremitus are the three trustworthy signs.

*Incision* in long-standing cases will be considered under the head of Empyema.

(*To be continued.*)

